# Impact of Platelet Count in Retinopathy of Prematurity

**DOI:** 10.4274/tjo.galenos.2020.01058

**Published:** 2020-12-29

**Authors:** Nedime Şahinoğlu Keşkek, Hande Gülcan, Gürsel Yılmaz, İmren Akkoyun

**Affiliations:** 1 Başkent University Faculty of Medicine, Adana Research and Training Center, Department of Ophthalmology, Adana, Turkey; 2 Başkent University Faculty of Medicine, Adana Research and Training Center, Department of Pediatrics, Division of Neonatology, Adana, Turkey; 3 Başkent University Faculty of Medicine, Department of Ophthalmology, Ankara, Turkey

**Keywords:** Birth weight, gestational age, retinopathy of prematurity, risk factors, platelet

## Abstract

**Objectives:**

The aim of the study was to investigate the risk factors for retinopathy of prematurity (ROP), including platelet count.

**Materials and Methods:**

This retrospective study analyzed 137 infants in 3 subgroups: no ROP; mild ROP, and severe ROP requiring laser treatment (type 1 ROP). A retrospective review of records was performed and statistical analysis of possible risk factors for ROP including platelet count was evaluated by using logistic regression.

**Results:**

Birth weight (BW), gestational age (GA), and low platelet count in the first week after birth were significant risk factors for developing ROP (p=0.038, 0.02, and 0.004, respectively). BW, GA, ventilation, and lower platelet count were associated with progression to type 1 ROP (p=0.004; 0.027, and 0.021, respectively).

**Conclusion:**

Lower platelet count in the first week after birth is a risk factor for ROP development in addition to the previously established factors of ventilation need, low BW, and low GA.

## Introduction

Retinopathy of prematurity (ROP) is a proliferating retinal vascular disorder that can result in poor vision in premature infants.^1^ The frequency of ROP-associated blindness is low in developed countries. However, in developing countries, the incidence of ROP-associated blindness is higher due to the increased survival of premature infants, a lack of standardized neonatal intensive care unit (NICU) conditions, and limited fundoscopic follow-up evaluations.^2,3^ The crucial risk factors for ROP development are low birth weight (BW) and low gestational age (GA).^4,5^ The other risk factors are oxygen therapy needs, sex, sepsis, patent ductus arteriosus (PDA), intraventricular hemorrhage, neonatal infections, necrotizing enterocolitis (NEC), and blood transfusion needs.^4,6,7,8,9,10^

Several studies have focused on the role of platelets in angiogenesis and hypothesized that thrombocytopenia might be a possible factor for developing ROP.^11,12^ Platelets accumulate, carry, and deliver distinct key regulators of angiogenesis such as vascular endothelial growth factor (VEGF), insulin-like growth factor-1 (IGF-1), and platelet-derived growth factor (PDGF).^13^ Low platelet count may cause delay in normal retinal vascularization and lead to subsequent unregulated retinal neovascularization due to lack of VEGF, IGF-1, and PDGF.^11,12^ Lundgren et al.^14^ noted that aggressive posterior ROP is associated with multiple infectious episodes and thrombocytopenia.

The purpose of this study was to investigate the impact of platelet count in ROP development beyond other known risk factors.

## Materials and Methods

The study included 137 infants with a GA of up to 34 weeks who were screened for ROP in the NICU of Başkent University in Adana, Turkey between July 2014 and July 2017. All infants with a GA of up to 34 weeks that were followed up until at least 43 weeks postconception were enrolled in the study (n=137). All the babies included in the study were born at the study site. Exclusion criteria were GA of more than 34 weeks (n=20) and lack of regular follow-up examinations at our institution until 43 weeks postconception (n=18). The Institutional Review Board of Başkent University Faculty of Medicine approved the study (KA:17/308). Informed consent was obtained from the parents of all infants included in the study.

ROP screening was performed by an experienced ophthalmologist (N.S.) using an indirect ophthalmoscope at the postnatal age of 4 weeks or PMA of 31 weeks according to screening guidelines.^15^ Follow-up examinations were conducted until retinal vascularization reached the ora serrata for 360°. Follow-up intervals were scheduled according to ROP severity. Infants with ROP were examined more often, according to the severity of the disorder. Phenylephrine 2.5% and tropicamide 0.5% were used for dilatation of the pupils. Fundus examination was conducted using an indirect ophthalmoscope and 28 D lens. Fundus findings were noted, and ROP was categorized according to the International Classification of Retinopathy of Prematurity.^15^

The infants were divided into 3 groups: Group A (no ROP) included babies without retinopathy, Group B (mild ROP) included babies diagnosed with stage 1 or stage 2 ROP but regressed, and Group C (severe ROP) included infants that progressed to Type 1 ROP and underwent laser treatment.^16^ We noted no noticeable asymmetry in any patient, and no eye progressed to stage 4 or 5 ROP.

We retrospectively reviewed the patient medical records from birth to 43 weeks of age for data such as GA, BW, sex, neonatal morbidities, respiratory distress syndrome (RDS), NEC, intraventricular hemorrhage, PDA, sepsis, red blood cell (RBC) transfusion, apnea, multiple pregnancy of the mother, ventilation need, and surfactant use for RDS. All available laboratory measurements of platelet counts were recorded. The values in the first postnatal week and within 1 week of ROP diagnosis were noted.

Univariate analysis was performed to reveal the significant risk factors for ROP, and the risk factors were included in the logistic regression. Ten potential risk factors (BW, GA, multiple pregnancy, ventilation need, RDS, NEC, PDA, RBC transfusion, apnea, sepsis, surfactant use, and platelet count in the first postnatal week) were analyzed with logistic regression to determine relationships between the variables and identify independent risk factors for ROP. Our criteria for dropping variables during backward stepwise logistic regression was p=0.05.

## Results

We evaluated 137 neonates in our NICU with GA ≤34 weeks during the study period. ROP was diagnosed in 47 cases (34.3%). Severe ROP was detected and treated in 15 cases (10.9%).

Univariate analysis showed that, in order of significance, BW, GA, PDA, RDS, RBC transfusion, apnea, low platelet count in the first postnatal week, ventilation need, surfactant, and sepsis were associated with ROP based on the p values. Risk factors are listed in Table 1.

With subsequent logistic regression analysis, BW, GA, and low platelet count in the first postnatal week were shown to be independent risk factors for ROP development. Also, logistic regression analysis demonstrated that GA, ventilation, and low platelet count in the first postnatal week were independently related to severe ROP. Low platelet count in the first postnatal week was shown to be an independent risk factor for both ROP development and progression.

The mean GA was 31.3±2.2 weeks for Group A, 28.5±1.8 weeks for Group B, and 27.4±1.5 weeks for Group C. The mean GA of Group A was significantly different from Group B and Group C (p<0.05 for each). Group B and C were not statistically significantly different (p=0.17).

Mean platelet count in the first postnatal week was 280±103x10^3^/µL in Group A, 222±69x10^3^/µL in Group B, and 214±62x10^3^/µL in Group C. According to the post hoc analysis, Group A had a significantly higher mean platelet count than Groups B and C (p=0.002). Group B and C were not statistically significantly different.

The mean platelet count in the week of ROP diagnosis was 339±147x10^3^/µL in Group B and 366±121x10^3^/µL in Group C. Group B and C were not statistically significantly different (p=0.5).

## Discussion

The incidence of ROP varies among countries due to different socioeconomic development and variability in study designs and survival rates. In the current study, the overall ROP incidence was 34.3%, while severe ROP was recorded in 10.9% of the infants. These results were similar to the rates of developing countries.^17,18,19^

In developing countries, infants with higher BW and GA are at risk for ROP development.^20,21^ Therefore, the current study consisted of infants with a GA ≤34 weeks. In a recent ROP study, fundus examinations of infants with GA ≤34 weeks or BW <1,700 g was recommended for Turkey.^22^

In our study, low GA, low BW, and low platelet count in the first week after birth were independent risk factors for developing ROP. However, low GA, ventilation need, and low platelet count in the first week after birth arose as independent risk factors for ROP progression.

Both low GA and low BW are associated with incomplete vascular and retinal neural development at birth, given the vulnerable structure of the retina.^4^ In our study, according to the logistic regression, GA was an independent risk factor for developing mild and severe ROP. However, BW was not statistically significant as a risk factor of severe ROP. This result may indicate the importance of weight gain to prevent the progression of ROP.^23,24,25^

The association between ROP and blood transfusion is well documented.^26^ The number of blood transfusions received by premature infants has been a major indicator of ROP in addition to GA and BW. Stutchfield et al.^26^ hypothesized that changing fetal hemoglobin to adult hemoglobin during transfusion may lead to ROP development by rapidly increasing oxygen accessibility to the retina. In our NICU, RBC transfusion was performed rather than whole blood transfusion when necessary. Therefore, RBC transfusion and platelet count were considered independent risk factors in our study. RBC transfusion was not found to be an independent risk factor for ROP, but this result may be due to the existence of many other risk factors in these infants.

Several pro- and antiangiogenic regulators were shown to be accumulated and carried in platelets.^11,12^ Platelet alpha granules have been shown to include IGF-1, IGF-binding protein 3 (the primary serum binding protein for IGF-1), VEGF, and platelet-derived growth factor. IGF-1 and VEGF levels are critical for ROP development.^27^ Our first hypothesis about the mechanism linking low platelet count and ROP development implies the delivery of IGF-1 by platelets. While IGF-1 is needed for VEGF-induced vessel growth, low platelet count at an early gestational week slows down vasculogenesis and leads to development of subsequent type 1 ROP.

ROP is a disorder with pathological angiogenesis in the inner retina and preretinal space.^28^ The newly formed blood vessels are not mature, which may lead to vascular leakage.^28^ Pericytes have a crucial role in angiogenesis by contributing survival signals for endothelial cells.^29^ PDGF is essential for pericyte viability.^30^ Moreover, PDGF is fundamental for both proliferation and migration of endothelial cells.^30^ A lack of pericytes is connected with endothelial hyperplasia, dilated capillaries, irregularly shaped endothelial cells, and increased transendothelial permeability.^30^ Hammes et al.^31^ indicated that PDGF-deficient mice had fewer pericytes compared to wild-type mice during the early postnatal phase of the growing retina. They studied a PDGF-receptor β-deficient mice model of oxygen-induced proliferative retinopathy (resembling ROP) to investigate the proliferative phase of diabetic retinopathy. PDGF-receptor β-deficient mice had significantly lower pericyte numbers and significantly higher numbers of acellular capillaries compared with wild-type. After exposure to a high-oxygen environment, the neovascular response to hypoxia nearly doubled in PDGF-receptor β-deficient mice. They also noted the degeneration of endothelial cells (indicated by narrow vessels) and obstructive occlusion in the absence of the PDGF-β receptor.^31^ Pericytes likely have a role in promoting endothelial cell survival and limiting endothelial hyperplasia. Our second hypothesis about the mechanism linking low platelet count and ROP development is the lack of PDGF. Our results and data from the literature demonstrate that at high VEGF levels (e.g., ROP), the deficiency of pericyte coverage due to low levels of circulating PDGF may lead to an increased neovascular response.

In ROP models, the introduction of hyperoxia to the retinas of newborn rats decreased VEGF levels and weakens retinal angiogenesis.^32,33^ Relative hypoxia of room air during the second week led to increased VEGF synthesis and pathological angiogenesis.^34^ During this proliferative phase of ROP, VEGF levels increase locally and systemically.^35^

VEGF induces endothelial cell migration and proliferation after hypoxia.^36^ During that period, thrombocytopenia may deepen the PDGF deficiency which is necessary for pericyte viability. PDGF deficiency may result in pathological angiogenesis.

Vinekar et al.^12^ presented a case of aggressive posterior ROP with severe thrombocytopenia regressing after serum platelet transfusions. Jensen et al.^11^ showed a relation between thrombocytopenia and the existence of type 1 ROP in zone 1 cases. The results of these studies suggest thrombocytopenia is a risk factor for zone 1 ROP. Cakir et al.^37^ showed that any episode of thrombocytopenia at ≥30 weeks postmenstrual age (PMA), was associated with severe ROP in a mouse model of ROP. The researchers evaluated mean weekly platelet count of mice and found a statistically significant difference between the severe ROP group and the no or less severe ROP group. On the contrary, Jensen et al.^38^ demonstrated that thrombocytopenia from birth to 34 weeks of PMA was related to severe ROP. In the current study, we evaluated the platelet count of the infants on the week of delivery and found lower platet count as a risk factor for ROP development. Our result is compatible with study by Jensen et al.^38^

The study group of the current study included all ROP cases classified in zone 1 and zone 2. Although platelet counts did not reach the level of thrombocytopenia and none of the infants needed a platelet transfusion, there was a significant difference in platelet count between infants that developed ROP and those who did not. Platelets are major regulators of angiogenic regulatory proteins such as VEGF and PDGF, which are stored, transported, and delivered by platelets.^13^ Our findings suggest that the growth factors in circulating platelets have a potential protective role against ROP and are necessary for retinal vascular maturation.

## Conclusion

The infants with lower platelet counts may have a higher risk for developing ROP. Our findings further contribute to the body of work producing a predictive model to estimate the likelihood for an infant to develop ROP. Further large-scale studies are required to define the potential relation between thrombocytopenia and ROP.

## Figures and Tables

**Table 1:**
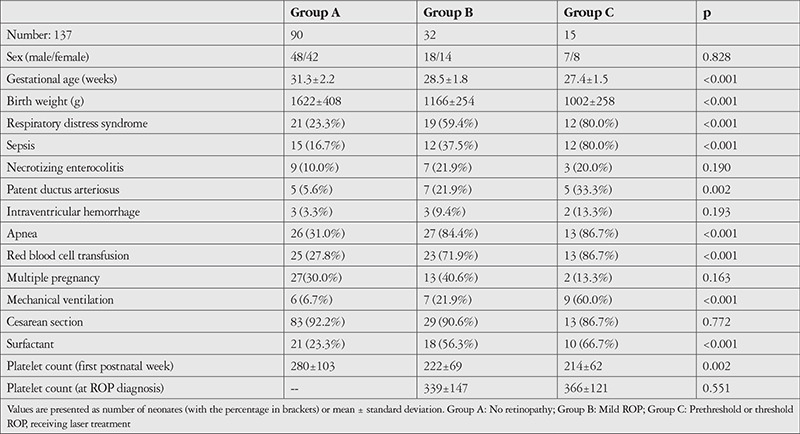
Comparison of the demographic characteristics and morbidities of infants with and without retinopathy of prematurity (ROP)
